# Dopamine Dysregulation Syndrome and Deep Brain Stimulation of the Subthalamic Nucleus in Parkinson's Disease

**DOI:** 10.1155/2011/759895

**Published:** 2011-11-02

**Authors:** Beatriz De la Casa-Fages, Francisco Grandas

**Affiliations:** ^1^Movement Disorders Research Unit, Hospital Universitario Gregorio Marañón, 28007 Madrid, Spain; ^2^Servicio de Neurología, Hospital General Universitario Gregorio Marañón, C/Doctor Esquerdo 46, 28007 Madrid, Spain

## Abstract

Dopamine dysregulation syndrome is a complication of the dopaminergic treatment in Parkinson's disease that may be very disabling due to the negative impact that compulsive medication use may have on patients' social, psychological, and physical functioning. The relationship between subthalamic nucleus deep brain stimulation and dopamine dysregulation syndrome in patients with Parkinson's disease remains unclear. Deep brain stimulation may improve, worsen, or have no effect on preoperative dopamine dysregulation syndrome. Moreover, dopamine dysregulation syndrome may appear for the first time after deep brain stimulation of the subthalamic nucleus. The outcome of postoperative dopamine dysregulation syndrome is poor despite stimulation and medication adjustments. Here we review the phenomenology and neurobiology of this disorder, discuss possible mechanisms that may underlie the diverse outcomes of dopamine dysregulation syndrome after subthalamic nucleus deep brain stimulation, and propose management strategies.

## 1. Dopamine Dysregulation Syndrome: Phenomenology, Epidemiology, and Risk Factors

Dopamine dysregulation syndrome (DDS), originally described as “hedonistic homeostatic dysregulation” by Giovannoni et al. [1], is a disturbance that may complicate long-term dopamine replacement therapy (DRT) of Parkinson's disease (PD). Patients with DDS develop an addictive pattern of DRT use, self-administering doses of dopaminergic drugs in excess of those required to control their motor symptoms. Patients demand drug escalation and a compulsive DRT seeking and intake is developed, taking larger levodopa doses than prescribed. They justify the overuse of antiparkinsonian drugs, despite being very dyskinetic, to avoid the nonmotor aspects related to “off” state such as anxiety, depressed mood, or fear sensation. Attempts made by physicians to reduce DRT doses are received with resistance and commonly are unsuccessful [2].

DDS has been related to levodopa and potent short-acting dopamine agonists as subcutaneous apomorphine, but it also can occur with other dopamine agonists. The compulsive use of DRT may lead to negative social behaviours such as hoarding medication.

DDS may encompass other psychomotor pathologies due to antiparkinsonian therapy as punding—complex meaningless stereotyped and repetitive motor behaviours—and impulse control disorders, such as pathological gambling, hypersexuality, compulsive shopping, and binge eating [2]. 

DDS is probably underdiagnosed because patients may not complain of their behaviour and physicians may forget to ask about that. There are no large epidemiological studies about the prevalence of DDS in the general PD population. The prevalence of DDS in patients attending Parkinson's disease centers is 3-4% [1, 3]. 

The DDS diagnostic criteria proposed by Giovannoni et al. [1] in his seminal paper were (a) PD with documented levodopa responsiveness; (b) need for increasing doses of DRT in excess of those normally required to relieve parkinsonian symptoms and signs; (c) pattern of pathological use: expressed need for increased DRT in the presence of excessive and significant dyskinesias despite being “on”, drug hoarding or drug seeking behaviour, unwillingness to reduce DRT, and absence of painful dystonias; (d) impairment in social or occupational functioning: fights, violent behaviour, loss of friends, absence from work, loss of job, legal difficulties, arguments or difficulties with family; (e) development of hypomaniac, maniac, or cyclothymic affective syndrome in relation to DRT; (f) development of a withdrawal state characterized by dysphoria, depression, irritability, and anxiety on reducing the level of DRT; (g) duration of disturbance of at least 6 months. 

 Pezzella et al. used the following selection criteria for DDS [3]: (a) PD with documented levodopa responsiveness; (b) need to increase doses of DRT beyond those normally required to relieve parkinsonian symptoms and signs; (c) pattern of pathological use of DRT (chronic medication abuse, excluding occasional dosage increase while experiencing a sudden off or preparing for a social event) and current mood disorders (depression, anxiety, hypomaniac state, euphoria), behavioural disorders (pathological gambling, obsessional shopping, hypersexuality, aggression, social isolation), or alteration of the perception of the on state (walkabouts, stereotypies).

Some factors may contribute or predispose to develop DDS in PD patients. 

Impulsivity and sensation and novelty-seeking personality traits have been found in patients with PD and DDS [4–6]. It has been also noticed that young-onset PD patients are more vulnerable to develop this syndrome [5]. Other reported risk factors are male gender, previous history of substance abuse (alcohol, drugs, etc.), higher dopaminergic drug intake, and presence of depressive symptoms [4–7]. Recently, engagement in a creative or artistic profession has been described as an additional risk factor for developing DDS [8]. 

Genetic factors may also be relevant [6]. It has been suggested that parkin gene mutations (PARK2) may be a risk factor for developing DDS [9, 10]. O'Sullivan et al. [4] proposed that “D2-like” receptor family genes could play a role in the pathogenesis of DDS. In PET studies with [11C]-raclopride, Parkin-positive medicated patients have been reported to show a significant decrease of D2 striatal receptors in comparison with idiopathic PD medicated patients and with age-matched healthy controls [10].

## 2. Neurobiology of DDS

Many PD patients with DDS fulfil DSM-IV criteria for substance dependence because of the negative effect that the pathological use of medication has on their social, psychological, and physical functioning [11]. 

The negative reinforcement model is one of the proposed theories to explain addiction [2]. Based on this model, patients with DDS may use dopaminergic drugs to avoid dysphoric off states. However, the model that may better explain the pathophysiology of DDS is the incentive-sensitization theory [4, 7]. This theory of addiction postulates that compulsive drug use modifies dopaminergic neurotransmission in the nucleus accumbens and related reward circuitry involving the mesocorticolimbic dopamine system [11]. This neuroadaptation on the accumbens-related circuitry makes the system sensitized to the psychomotor effects of drugs [7]. Thus, the potential addictive pattern of antiparkinsonian drugs may be related to their effect on the reward system. Some experimental data on the activation of accumbens-related reward-seeking pathways by DRT suggested that antiparkinsonian drugs share some properties with addictive psychostimulants as amphetamine [7]. 

There is evidence of sensitization of the ventral striatum reward system by PET imaging with [11C]-raclopride in PD patients with DDS who had enhanced levodopa-induced ventral striatal dopamine release compared with levodopa-treated patients with no compulsive drug use [11]. Thus, PD patients with more pronounced mesolimbic dopaminergic denervation, involving the ventral striatum (nucleus accumbens), may be prone to develop compulsive behaviours after dopaminergic treatment [11, 12].

## 3. Dopamine Dysregulation Syndrome and Subthalamic Nucleus Deep Brain Stimulation

Deep brain stimulation of the subthalamic nucleus (STN-DBS) is an established therapy for advanced PD patients with motor complications [13]. Bilateral continuous high-frequency stimulation of the STN improves motor disability by 33–67%, motor fluctuations by 73–83%, and levodopa-induced dyskinesias by 55–88% and permits a 40–80% reduction in the doses of antiparkinsonian medication, compared with the preoperative state [14]. However, adverse neurocognitive, mood, and behavioral changes may appear after STN-DBS [15].

Despite the motor improvement of patients who underwent STN-DBS, its effect on nonmotor symptoms of Parkinson's disease, especially neurobehavioral disorders as impulse control disorders, punding, and DDS has not been widely studied.

The relationship between STN-DBS and DDS in patients with Parkinson's disease remains unclear. While some authors described a complete resolution of the behaviour disorder after STN-DBS [16–19], others found no improvement or even worsening of DDS postoperatively [18]. Furthermore, de novo DDS may appear as a complication of STN-DBS [14, 19–21].

### 3.1. Dopamine Dysregulation Syndrome Improved or Resolved after DBS

To our knowledge, there are 9 PD patients with DDS reported in the literature who improved or resolved after STN-DBS [16–19]. Witjas et al. described two patients with preoperative DDS that resolved immediately after surgery without recurrence of the compulsive medication use in successive years [16]. Knobel et al. described a PD patient suffering from severe DDS necessitating in-ward psychiatric management, who, following STN-DBS and medication reduction, had a rapid and dramatic resolution of DDS and associated psychiatric symptoms [17]. Bandini et al. reported one patient who had also a great improvement of DDS within the first month after surgery [18]. Lim and colleagues described three resolved DDS cases after STN-DBS and other two patients that improved their behaviour after the procedure [19].

Postoperative DDS improvement may be related to the reduction of dopaminergic agents allowed by surgery [16, 19, 22]. Decreasing the pulsatile administration of levodopa may decrease the experience of negative emotional states and reduce the abnormal sensitization of motivational symptoms associated with the mesolimbic system [7]. However this argument cannot be applied in all cases [19].

### 3.2. DDS Remained Unchanged or Worsened after DBS

Subthalamic nucleus deep brain stimulation may not influence DDS evolution or even can worsen it. 71% of patients with preoperative DDS reported by Lim et al. (12 patients) remained unimproved or worsened after surgery [19].

### 3.3. De Novo DDS after DBS

Interestingly, dopamine dysregulation syndrome can also appear for the first time after deep brain stimulation of the subthalamic nucleus. We reviewed the literature searching for reports of PD patients in whom DDS appeared within 12 months of STN-DBS. To our knowledge, at least, 7 cases have been reported [14, 19–21]. 

The first patient with postoperative DDS was reported by Houeto et al. in 2002 [14], a 61-year-old man with personal history of alcohol misuse and bipolar disorder before the onset of the disease. The motor outcome after surgery was good (UPDRS III improved by 86%), although daily levodopa dose was reduced only by 12%. Diverse medications, stimulation adjustments, and psychotherapy did not resolve DDS. Three years later, the same group published a five-year followup of 37 PD patients treated with STN-DBS in their center [20]. They reported 3 patients addicted to levodopa treatment as permanent adverse effect related to medical treatment, bilateral STN-DBS, or progression of the disease [20]. We assume all these patients presented the DDS for the first time after surgery. Probably the case described by Houeto et al. [14] is one of the three cases reported.

Lim and colleagues reported in 2009 two patients with DDS which apparently appeared for the first time after bilateral STN-DBS [19]. However, one of the reported cases is not considered in this paper since DDS occurred 8 years after surgery in the context of a battery failure. 

We have recently reported three PD patients who developed DDS within a year after surgery [21]. The first case was a 47-year-old woman who underwent STN-DBS for a 10-year history of PD complicated with motor fluctuations and disabling dyskinesias. Neuropsychological exam prior to surgery was unremarkable. She had been treated with different antiparkinsonian drugs, including subcutaneous infusion of apomorphine. After DBS, off periods were significantly reduced, dyskinesias decreased, and medical treatment was reduced by 50% (apomorphine infusion was stopped). Six months after surgery, despite her motor improvement, her husband reported a compulsive intake of levodopa. The patient justified the overuse because “she felt herself as empty of energy.” In addition, she exhibited binge eating. Therapeutic approaches included pramipexole withdrawal, DBS adjustments, and addition of quetiapine and lorazepam, but five years after surgery she continued to overuse levodopa. The second case was a 59-year-old man with a 7-year history of PD complicated with motor fluctuations and mild depressive symptoms. Before surgery he was treated with high doses of levodopa plus multiple boluses of subcutaneous apomorphine. Despite the clinical motor improvement after STN-DBS, four months after surgery, the patient kept injecting himself apomorphine to avoid anxiety, depressive mood, and fear sensation. Addition of quetiapine, citalopram, and DBS parameter adjustments did not resolve the behaviour, and DDS persisted four years after surgery. The third patient was a 45-year-old man with PD since the age of 30, without previous psychiatric history who underwent STN-DBS surgery for severe motor fluctuations. A month after STN-DBS procedure, he started drinking high quantity of caffeine-rich beverages (coke) and developed hypersexuality. Two months later he admitted taking double dose of pramipexole than prescribed because he felt he “needed” it. Citalopram and quetiapine were prescribed and pramipexole tapered. Hypersexuality and compulsive coke drinking disappeared, but the patient became apathetic and began to overuse levodopa. In these cases DBS disconnection was tried, but switching off DBS led to rapid deterioration of their motor function in a matter of hours, a situation which was not tolerated by the patients.

A definite causal relationship with STN-DBS cannot be made since the outcome of DDS after discontinuing DBS could not be assessed—our patients did not tolerate it for the rapid worsening of their motor performance. Besides, DDS could have appeared in the evolution of the patients' disease independently of the surgical treatment. However, the temporal relationship between STN-DBS surgery and the onset of DDS suggests that STN-DBS might have played a role in the appearance of DDS.

One possible explanation for the appearance or worsening of DDS after STN-DBS could be misplacement of the electrodes in the medial zone of the STN, which corresponds to its limbic territory [23] and is related to motivational and emotional aspects of behavior [23, 24]. This explanation, however, seems unlikely since in all reported patients the motor outcome of the surgery was as expected after stimulation of the dorsolateral sensorimotor part of the STN [14]. However, due to the small size of the STN, stimulation with electrode contacts located mainly within the sensorimotor territory can result in the spread of current to associative and limbic areas as well as to surrounding structures [25]. Computational modelling to measure the volume of activated tissue during DBS is currently being investigated [25, 26].

Several neuroimaging studies have found that STN-DBS induced metabolic modifications in cortical and subcortical structures related to limbic and associative circuits [26–28]. Thus, the spread of stimulation to associative and limbic areas of the STN may underlie the appearance of DDS after DBS surgery. Impulse control disorders such as pathological gambling have also been reported after DBS [29, 30] as well as impulsive behaviour during high-conflict decisions [31] and apathy [32]. Indeed, besides DDS, most of the patients developed other behavioural addictions such as pathological gambling and abnormal sexual behaviour [14], binge eating [21], punding, and compulsive shopping [19]. 

On the other hand, the psychostimulant effect of STN-DBS [33, 34] seems an unlikely explanation for de novo DDS after surgery.

The seemingly paradoxical occurrence of either improvement or worsening of DDS after STN-DBS or, furthermore, the de novo appearance of this addictive behaviour may be related to individual differences in the extent of mesolimbic dopaminergic denervation [22, 35]. PET studies comparing PD patients with postoperative apathy after dopamine agonist withdrawal with nonapathetic patients have revealed areas of increased [11C]-raclopride binding potential values in the orbitofrontal, dorsolateral, prefrontal, and posterior cingulate cortices as well as other subcortical structures including the amygdala and the ventral striatum bilaterally in the apathetic group, thus suggesting the existence of different nonmotor phenotypes [22]. 

Reward-seeking behavior is mediated by the amygdala and nucleus accumbens, both of which receive dopaminergic projections from the ventral tegmental area [36]. Thus, PD patients with more pronounced mesolimbic dopaminergic denervation involving the ventral striatum (nucleus accumbens) may be prone to developing compulsive behaviours after dopaminergic treatment [5, 12]. 

This might be the case of de novo DDS patients, in whom the combined effect of dopaminergic replacement therapy (albeit reduced after surgery) and DBS on the limbic territory of the STN could have led to hyperstimulation of the mesolimbic system, thus precipitating the onset of DDS.

## 4. Management of Dopamine Dysregulation Syndrome in the Context of STN-DBS

The clinical management of DDS is challenging, particularly when appears after STN-DBS. Despite a good postoperative motor outcome, most patients overused dopaminergic drugs not because they felt pleasurable effects but to avoid anxiety, dysphoria, and other nonmotor symptoms [2, 21, 37]. 

Preoperative recognition of DDS and other impulse control disorders is correlated with a better outcome of these behaviours after DBS, whereas lack of recognition, poor motor benefit, and higher dopaminergic medication doses after surgery were associated with persistence or even new cases of DDS [19].

Changes in stimulation parameters, attempts to reduce the dose of dopaminergic drugs, and the addition of antipsychotics and/or antidepressants may not be helpful. The outcome of DDS after STN-DBS was poor in the majority of reported cases [14, 19–21].

The issue of how to manage antiparkinsonian drugs after STN-DBS remains unsettled. A large and rapid reduction in dopamine replacement therapy with withdrawal of dopaminergic agonists may induce apathy and severe depression in many patients [22], whereas a more conservative approach may precipitate DDS in some cases. 

Identifying vulnerable patients to develop DDS seems of paramount relevance. Young-onset PD, male gender, previous history of substance abuse, impulsive sensation seeking personality, presence of depressive symptoms, and artistic professions have been considered risk factors for DDS [4–8] as well as treatment with high doses of dopaminergic medication and rapid acting dopaminergic drugs [4–7]. Perhaps detailed neuropsychological evaluations using scales such as *QUIP (Questionnaire for Impulsive and Compulsive Disorders in Parkinson's disease)* [38] or PET studies to detect cases of severe denervation of the mesolimbic dopaminergic system [22] may be useful tools to identify potential DDS patients.

The adequate location of electrodes is another crucial factor. Contacts close to the ventromedial part of the STN should be avoided. Perhaps the use of computational modelling [25, 26], when it becomes available, could help elucidate the best set of stimulation parameters for individual patients. 

Nevertheless, it seems advisable to avoid a hyperdopaminergic state during the postoperative period, since high doses of dopaminergic medication added to STN-DBS therapy may predispose to developing DDS in vulnerable patients. 

Further research should be undertaken in order to identify patients who are vulnerable to DDS and to define the most appropriate postoperative management.
